# Supplementary material for: A Systematic Evaluation of High-Throughput Sequencing Approaches to Identify Low-Frequency Single Nucleotide Variants in Viral Populations
Source: Viruses. 2020 Oct 20;12(10):1187. doi: 10.3390/v12101187 (PMC7594041; doi:10.3390/v12101187)
Supplement: Supplementary file 1 [file viruses-12-01187-s001.zip › Supplementary figures.docx]

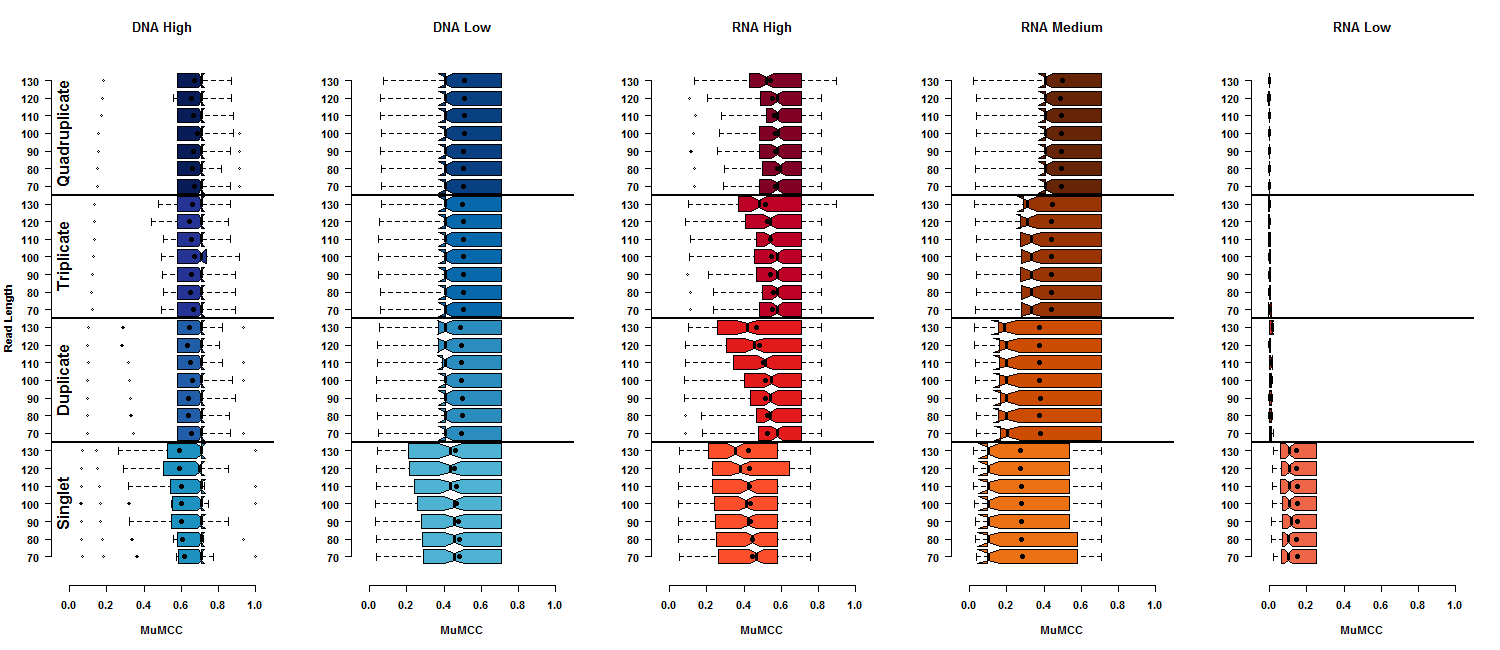


Supplementary figure S1. The effect of read length and number of replicates on variant calling accuracy.

The range of MuMCC scores for each population input for each read length tested following read alignment using GEM3 and selected qScore parameters. Singlet, duplicate, triplicate and quadruplicate technical replicates are represented by a different shade of colour. The solid black dot within each boxplot indicates the mean of the MuMCC distribution.
