# Supplementary material for: A Systematic Evaluation of High-Throughput Sequencing Approaches to Identify Low-Frequency Single Nucleotide Variants in Viral Populations
Source: Viruses. 2020 Oct 20;12(10):1187. doi: 10.3390/v12101187 (PMC7594041; doi:10.3390/v12101187)
Supplement: Supplementary file 1 [file viruses-12-01187-s001.zip › Supplementary tables.docx]

Supplementary table S1. A list of all programs and associated parameters pipelines tested.

|  |  |  |  |
| --- | --- | --- | --- |
|  | Program options | Program code | Parameter options |
| Sickle | qScore | -q | 0, 10, 20, 25, 30, 35, 38 |
|  | Read Length | -l | 70, 80, 90, 100, 110, 120, 130 |
| BWA-MEM | Minimum Seed Length | -k | 10, 20 |
|  | Re-seeding | -r | 1, 1.5, 2 |
| GEM3 | Mapping mode | --mapping-mode | fast, sensitive |
|  | Maximum alignment error | --alignment-max-error | 0.05, 0.10, 0.12, 0.15 |
| Bowtie2 | Local alignment | -- | very-fast-local, fast-local, sensitive-local, very-sensitive-local |
|  | Global alignment | -- | very-fast, fast, sensitive, very-sensitive |
| HiSAT2 | Most distinct primary alignment | -x | 0, 1, 3, 5, 7, 10, 13, 15 |
|  | Maximum seed length | -k | 1, 3, 5, 7, 10, 13, 15, 17, 20 |
| Smalt | Length of k-mer word index | -k | 11, 13, 16, 18, 20 |
|  | Sample every # k-mer | -s | 1, 3, 5, 7, 8, 11, 13, 16, 18, 20 |

| Post-alignment parameters | |
| --- | --- |
| Percentage frequency cut-off | 0.01%, 0.02%, 0.03%, 0.04%, 0.05%, 0.06%, 0.07%, 0.08%, 0.09%, 0.1%, 0.2%, 0.3%, 0.4%, 0.5%, 0.6%, 0.7%, 0.8%, 0.9%, 1% |
|  |  |

Supplementary table S2. The percentage frequency of raw reads from all DNA and RNA population types remaining following trimming using the different qScore and read length parameters tested in Sickle.

|  |  |  |  | qScore | | | | | | Read length | | | | | | | |
| --- | --- | --- | --- | --- | --- | --- | --- | --- | --- | --- | --- | --- | --- | --- | --- | --- | --- |
|  |  | Replicate | Total reads | >10 | >20 | >25 | >30 | >35 | >38 |  | >70 | >80 | >90 | >100 | >110 | >120 | >130 |
| RNA Swarm | High | 1 | 1.93x10^6^ | 99.34% | 98.27% | 94.49% | 86.90% | 70.60% | 42.31% |  | 82.79% | 77.67% | 72.61% | 67.71% | 62.99% | 58.30% | 53.83% |
|  |  | 2 | 2.22x10^6^ | 99.32% | 98.32% | 94.94% | 87.94% | 72.31% | 44.34% |  | 82.24% | 76.95% | 71.79% | 66.80% | 61.99% | 57.25% | 52.70% |
|  |  | 3 | 2.05x10^6^ | 99.38% | 98.37% | 94.97% | 87.90% | 72.11% | 43.66% |  | 82.81% | 77.62% | 72.52% | 67.58% | 62.80% | 58.09% | 53.57% |
|  |  | 4 | 1.08x10^6^ | 99.25% | 97.60% | 92.34% | 82.96% | 64.67% | 35.58% |  | 79.92% | 74.50% | 69.16% | 63.99% | 59.11% | 54.27% | 49.68% |
|  | Medium | 1 | 1.87x10^6^ | 99.33% | 98.36% | 94.84% | 87.63% | 71.82% | 43.63% |  | 82.85% | 77.78% | 72.79% | 67.92% | 63.27% | 58.62% | 54.16% |
|  |  | 2 | 1.97x10^6^ | 99.38% | 98.46% | 95.28% | 88.66% | 73.60% | 46.24% |  | 84.13% | 79.44% | 74.80% | 70.30% | 65.91% | 61.43% | 57.17% |
|  |  | 3 | 1.91x10^6^ | 99.38% | 98.46% | 95.36% | 88.68% | 73.31% | 45.06% |  | 83.35% | 78.38% | 73.50% | 68.73% | 64.16% | 59.56% | 55.21% |
|  |  | 4 | 4.80x10^6^ | 98.65% | 96.74% | 91.23% | 81.69% | 63.35% | 34.38% |  | 71.04% | 63.84% | 57.12% | 50.96% | 45.45% | 40.33% | 35.46% |
|  | Low | 1 | 2.12x10^6^ | 99.40% | 98.28% | 94.32% | 86.78% | 70.78% | 42.82% |  | 83.42% | 78.46% | 73.54% | 68.73% | 64.11% | 59.50% | 55.06% |
|  |  | 2 | 2.16x10^6^ | 99.46% | 98.37% | 94.75% | 87.58% | 72.04% | 44.29% |  | 83.56% | 78.66% | 73.83% | 69.04% | 64.42% | 59.84% | 55.40% |
|  |  | 3 | 1.82x10^6^ | 99.46% | 98.37% | 94.69% | 87.33% | 71.38% | 43.13% |  | 82.43% | 77.06% | 71.75% | 66.62% | 61.74% | 56.89% | 52.28% |
|  |  | 4 | 1.96x10^6^ | 99.30% | 97.55% | 91.95% | 82.56% | 64.60% | 35.98% |  | 80.29% | 74.95% | 69.77% | 64.78% | 60.02% | 55.33% | 50.79% |
| DNA Swarm | High | 1 | 1.60x10^6^ | 99.39% | 98.24% | 94.21% | 86.35% | 69.64% | 41.07% |  | 82.21% | 76.92% | 71.73% | 66.71% | 61.88% | 57.09% | 52.53% |
|  |  | 2 | 1.06x10^6^ | 99.43% | 98.38% | 94.84% | 87.71% | 71.90% | 43.79% |  | 83.43% | 78.52% | 73.70% | 68.98% | 64.39% | 59.80% | 55.40% |
|  |  | 3 | 2.14x10^6^ | 99.35% | 98.28% | 94.68% | 87.35% | 71.17% | 42.58% |  | 81.93% | 76.53% | 71.28% | 66.20% | 61.32% | 56.54% | 51.96% |
|  |  | 4 | 2.56x10^6^ | 99.26% | 97.45% | 91.78% | 82.09% | 63.67% | 34.83% |  | 78.50% | 72.65% | 67.06% | 61.66% | 56.56% | 51.55% | 46.84% |
|  | Low | 1 | 1.84x10^6^ | 99.12% | 97.27% | 91.73% | 82.95% | 66.03% | 38.91% |  | 77.99% | 72.08% | 66.38% | 60.92% | 55.82% | 50.85% | 46.16% |
|  |  | 2 | 2.25x10^6^ | 99.13% | 97.48% | 92.54% | 84.11% | 67.43% | 40.04% |  | 78.43% | 72.53% | 66.78% | 61.25% | 55.53% | 50.96% | 46.18% |
|  |  | 3 | 2.38x10^6^ | 99.03% | 97.22% | 92.00% | 83.28% | 66.43% | 38.93% |  | 76.86% | 70.64% | 64.66% | 58.99% | 53.71% | 48.57% | 43.78% |
|  |  | 4 | 2.67x10^6^ | 98.84% | 96.03% | 88.56% | 77.95% | 59.53% | 32.24% |  | 73.21% | 66.78% | 60.69% | 54.95% | 49.58% | 44.43% | 39.63% |

Supplementary table S3a. The mean coverage range across the sequenced amplicon for every parameter tested for each short-read aligner for all DNA samples.

|  |  |  | BWA-MEM | GEM3 | Bowtie2-Local | Bowtie2-Global | HiSAT2 | SMALT |
| --- | --- | --- | --- | --- | --- | --- | --- | --- |
| DNA *High* | 1 | Minimum | 3.25x10^3^ | 3.25x10^3^ | 3.25x10^3^ | 3.15x10^3^ | 3.16x10^3^ | 3.25x10^3^ |
|  |  | Maximum | 4.73x10^4^ | 4.71x10^4^ | 4.73x10^4^ | 4.64x10^4^ | 4.62x10^4^ | 4.72x10^4^ |
|  |  | Mean | 2.77x10^4^ | 2.76x10^4^ | 2.76x10^4^ | 2.71x10^4^ | 2.71x10^4^ | 2.76x10^4^ |
|  |  | Median | 2.85x10^4^ | 2.84x10^4^ | 2.84x10^4^ | 2.80x10^4^ | 2.79x10^4^ | 2.85x10^4^ |
|  | 2 | Minimum | 2.40x10^3^ | 2.39x10^3^ | 2.39x10^3^ | 2.33x10^3^ | 2.34x10^3^ | 2.39x10^3^ |
|  |  | Maximum | 3.25x10^4^ | 3.24x10^4^ | 3.25x10^4^ | 3.19x10^4^ | 3.18x10^4^ | 3.25x10^4^ |
|  |  | Mean | 1.96x10^4^ | 1.96x10^4^ | 1.96x10^4^ | 1.92x10^4^ | 1.93x10^4^ | 1.96x10^4^ |
|  |  | Median | 2.03x10^4^ | 2.03x10^4^ | 2.03x10^4^ | 2.00x10^4^ | 2.00x10^4^ | 2.03x10^4^ |
|  | 3 | Minimum | 4.30x10^3^ | 4.29x10^3^ | 4.29x10^3^ | 4.16x10^3^ | 4.18x10^3^ | 4.29x10^3^ |
|  |  | Maximum | 6.27x10^4^ | 6.25x10^4^ | 6.27x10^4^ | 6.15x10^4^ | 6.16x10^4^ | 6.27x10^4^ |
|  |  | Mean | 3.71x10^4^ | 3.72x10^4^ | 3.71x10^4^ | 3.64x10^4^ | 3.67x10^4^ | 3.71x10^4^ |
|  |  | Median | 3.79x10^4^ | 3.78x10^4^ | 3.78x10^4^ | 3.72x10^4^ | 3.74x10^4^ | 3.78x10^4^ |
|  | 4 | Minimum | 3.69x10^3^ | 3.68x10^3^ | 3.68x10^3^ | 3.52x10^3^ | 3.59x10^3^ | 3.68x10^3^ |
|  |  | Maximum | 7.05x10^4^ | 7.02x10^4^ | 7.05x10^4^ | 6.92x10^4^ | 6.93x10^4^ | 7.05x10^4^ |
|  |  | Mean | 3.83x10^4^ | 3.77x10^4^ | 3.82x10^4^ | 3.76x10^4^ | 3.72x10^4^ | 3.82x10^4^ |
|  |  | Median | 3.93x104 | 3.78x10^4^ | 3.92x10^4^ | 3.86x10^4^ | 3.73x10^4^ | 3.93x10^4^ |
| DNA *Low* | 1 | Minimum | 3.75x10^3^ | 4.99x10^4^ | 3.75x10^3^ | 3.64x10^3^ | 3.65x10^3^ | 3.75x10^3^ |
|  |  | Maximum | 5.01x10^4^ | 3.74x10^3^ | 5.02x10^4^ | 4.92x10^4^ | 4.91x10^4^ | 5.01x10^4^ |
|  |  | Mean | 2.82x10^4^ | 2.80x10^4^ | 2.82x10^4^ | 2.77x10^4^ | 2.75x10^4^ | 2.82x10^4^ |
|  |  | Median | 2.83x10^4^ | 2.80x10^4^ | 2.83x10^4^ | 2.78x10^4^ | 2.76x10^4^ | 2.83x10^4^ |
|  | 2 | Minimum | 4.49x10^3^ | 4.48x10^3^ | 4.48x10^3^ | 4.36x10^3^ | 4.38x10^3^ | 4.48x10^3^ |
|  |  | Maximum | 6.11x10^4^ | 6.09x10^4^ | 6.11x10^4^ | 6.00x10^4^ | 5.97x10^4^ | 6.11x10^4^ |
|  |  | Mean | 3.47x10^4^ | 3.46x10^4^ | 3.47x10^4^ | 3.41x10^4^ | 3.40x10^4^ | 3.47x10^4^ |
|  |  | Median | 3.55x10^4^ | 3.47x10^4^ | 3.54x10^4^ | 3.49x10^4^ | 3.42x10^4^ | 3.55x10^4^ |
|  | 3 | Minimum | 4.35x10^3^ | 4.34x10^3^ | 4.34x10^3^ | 4.21x10^3^ | 4.23x10^3^ | 4.34x10^3^ |
|  |  | Maximum | 6.23x10^4^ | 6.20x10^4^ | 6.23x10^4^ | 6.11x10^4^ | 6.11x10^4^ | 6.23x10^4^ |
|  |  | Mean | 3.48x10^4^ | 3.46x10^4^ | 3.48x10^4^ | 3.42x10^4^ | 3.41x10^4^ | 3.48x10^4^ |
|  |  | Median | 3.58x10^4^ | 3.46x10^4^ | 3.57x10^4^ | 3.51x10^4^ | 3.41x10^4^ | 3.57x10^4^ |
|  | 4 | Minimum | 3.63x10^3^ | 3.62x10^3^ | 3.63x10^3^ | 3.52x10^3^ | 3.51x10^3^ | 3.63x10^3^ |
|  |  | Maximum | 6.62x10^4^ | 6.58x10^4^ | 6.63x10^4^ | 6.47x10^4^ | 6.47x10^4^ | 6.62x10^4^ |
|  |  | Mean | 3.41x10^4^ | 3.33x10^4^ | 3.41x10^4^ | 3.34x10^4^ | 3.33x10^4^ | 3.41x10^4^ |
|  |  | Median | 3.31x10^4^ | 3.27x10^4^ | 3.31x10^4^ | 3.25x10^4^ | 3.24x10^4^ | 3.31x10^4^ |

Supplementary table S3b. The mean coverage range across the sequenced amplicon for every parameter tested for each short-read aligner for all RNA samples.

|  |  |  | BWA-MEM | GEM3 | Bowtie2-Local | Bowtie2-Global | HiSAT2 | SMALT |
| --- | --- | --- | --- | --- | --- | --- | --- | --- |
| RNA *High* | 1 | Minimum | 4.24x10^3^ | 4.23x10^3^ | 4.23x10^3^ | 4.11x10^3^ | 4.15x10^4^ | 3.25x10^4^ |
|  |  | Maximum | 5.78x10^4^ | 5.76x10^4^ | 5.78x10^4^ | 5.67x10^4^ | 5.83x10^4^ | 4.72x10^4^ |
|  |  | Mean | 3.43x10^4^ | 3.42x10^4^ | 3.42x10^4^ | 3.36x10^4^ | 3.41x10^4^ | 2.76x10^4^ |
|  |  | Median | 4.74x10^4^ | 3.53x10^4^ | 3.53x10^4^ | 3.87x10^4^ | 3.51x10^4^ | 2.85x10^4^ |
|  | 2 | Minimum | 5.08x10^3^ | 5.07x10^3^ | 5.07x10^3^ | 4.92x10^3^ | 4.94x10^3^ | 5.07x10^3^ |
|  |  | Maximum | 6.53x10^4^ | 6.50x10^4^ | 6.53x10^4^ | 6.40x10^4^ | 6.39x10^4^ | 6.52x10^4^ |
|  |  | Mean | 3.93x10^4^ | 3.94x10^4^ | 3.92x10^4^ | 3.85x10^4^ | 3.88x10^4^ | 3.93x10^4^ |
|  |  | Median | 4.04x10^4^ | 4.03x10^4^ | 4.03x10^4^ | 3.96x10^4^ | 3.97x10^4^ | 4.04x10^4^ |
|  | 3 | Minimum | 4.43x10^3^ | 4.42x10^3^ | 4.42x10^3^ | 4.29x10^3^ | 4.31x10^3^ | 4.42x10^3^ |
|  |  | Maximum | 6.11x10^4^ | 6.09x10^4^ | 6.11x10^4^ | 5.99x10^4^ | 5.98x10^4^ | 6.11x10^4^ |
|  |  | Mean | 3.69x10^4^ | 3.67x10^4^ | 3.65x10^4^ | 3.59x10^4^ | 3.61x10^4^ | 3.66x10^4^ |
|  |  | Median | 3.74x10^4^ | 3.73x10^4^ | 2.29x10^4^ | 3.67x10^4^ | 3.67x10^4^ | 3.74x10^4^ |
|  | 4 | Minimum | 1.65x10^3^ | 1.65x10^3^ | 1.65x10^3^ | 1.60x10^3^ | 1.61x10^3^ | 1.65x10^3^ |
|  |  | Maximum | 3.10x10^4^ | 3.09x10^4^ | 3.11x10^4^ | 3.05x10^4^ | 3.03x10^4^ | 3.10x10^4^ |
|  |  | Mean | 1.71x10^4^ | 1.69x10^4^ | 1.71x10^4^ | 1.68x10^4^ | 1.66x10^4^ | 1.71x10^4^ |
|  |  | Median | 1.80x10^4^ | 1.71x10^4^ | 1.79x10^4^ | 1.77x10^4^ | 1.69x10^4^ | 1.80x10^4^ |
| RNA *Medium* | 1 | Minimum | 4.40x10^3^ | 4.39x10^3^ | 4.39x10^3^ | 4.27x10^3^ | 4.28x10^3^ | 4.39x10^3^ |
|  |  | Maximum | 5.62x10^4^ | 5.61x10^4^ | 5.62x10^4^ | 5.52x10^4^ | 5.53x10^4^ | 5.62x10^4^ |
|  |  | Mean | 3.38x10^4^ | 3.39x10^4^ | 3.38x10^4^ | 3.32x10^4^ | 3.34x10^4^ | 3.38x10^4^ |
|  |  | Median | 3.47x10^4^ | 3.47x10^4^ | 3.47x10^4^ | 3.41x10^4^ | 3.42x10^4^ | 3.47x10^4^ |
|  | 2 | Minimum | 5.44x10^3^ | 5.43x10^3^ | 5.42x10^3^ | 5.28x10^3^ | 5.29x10^3^ | 5.43x10^3^ |
|  |  | Maximum | 6.14x10^4^ | 6.12x10^4^ | 6.13x10^4^ | 6.02x10^4^ | 6.03x10^4^ | 6.13x10^4^ |
|  |  | Mean | 3.80x10^4^ | 3.82x10^4^ | 3.80x10^4^ | 3.73x10^4^ | 3.76x10^4^ | 3.80x10^4^ |
|  |  | Median | 3.92x10^4^ | 3.91x10^4^ | 3.91x10^4^ | 3.85x10^4^ | 3.85x10^4^ | 3.92x10^4^ |
|  | 3 | Minimum | 4.44x10^3^ | 4.43x10^3^ | 4.43x10^3^ | 4.30x10^3^ | 4.31x10^3^ | 4.43x10^3^ |
|  |  | Maximum | 5.79x10^4^ | 5.77x10^4^ | 5.79x10^4^ | 5.69x10^4^ | 5.67x10^4^ | 5.79x10^4^ |
|  |  | Mean | 3.54x10^4^ | 3.55x10^4^ | 3.53x10^4^ | 3.47x10^4^ | 3.50x10^4^ | 3.53x10^4^ |
|  |  | Median | 3.63x10^4^ | 3.63x10^4^ | 3.62x10^4^ | 3.56x10^4^ | 3.57x10^4^ | 3.63x10^4^ |
|  | 4 | Minimum | 5.55x10^2^ | 5.53x10^2^ | 5.53x10^2^ | 5.30x10^2^ | 5.32x10^2^ | 5.53x10^2^ |
|  |  | Maximum | 1.07x10^4^ | 1.06x10^4^ | 1.07x10^4^ | 1.04x10^4^ | 1.04x10^4^ | 1.07x10^4^ |
|  |  | Mean | 5.58x10^3^ | 5.53x10^3^ | 5.57x10^3^ | 5.42x10^3^ | 5.40x10^3^ | 5.58x10^3^ |
|  |  | Median | 5.51x10^3^ | 5.46x10^3^ | 5.48x10^3^ | 5.32x10^3^ | 5.29x10^3^ | 5.51x10^3^ |
| RNA *Low* | 1 | Minimum | 4.94x10^3^ | 4.93x10^3^ | 4.93x10^3^ | 4.79x10^3^ | 4.81x10^3^ | 4.93x10^3^ |
|  |  | Maximum | 6.36x10^4^ | 6.34x10^4^ | 6.36x10^4^ | 6.24x10^4^ | 6.22x10^4^ | 6.36x10^4^ |
|  |  | Mean | 3.80x10^4^ | 3.79x10^4^ | 3.79x10^4^ | 3.73x10^4^ | 3.73x10^4^ | 3.80x10^4^ |
|  |  | Median | 3.94x10^4^ | 3.92x10^4^ | 3.93x10^4^ | 3.86x10^4^ | 3.86x10^4^ | 3.93x10^4^ |
|  | 2 | Minimum | 5.35x10^3^ | 5.34x10^3^ | 5.33x10^3^ | 5.23x10^3^ | 5.23x10^3^ | 5.34x10^3^ |
|  |  | Maximum | 6.64 10^4^ | 6.62x10^4^ | 6.63x10^4^ | 6.53x10^4^ | 6.55x10^4^ | 6.63x10^4^ |
|  |  | Mean | 4.01x10^4^ | 4.02x10^4^ | 4.00x10^4^ | 3.95x10^4^ | 3.98x10^4^ | 4.01x10^4^ |
|  |  | Median | 4.10x10^4^ | 4.10x10^4^ | 4.09x10^4^ | 4.05x10^4^ | 4.06x10^4^ | 4.10x10^4^ |
|  | 3 | Minimum | 3.94x10^3^ | 3.94x10^3^ | 3.94x10^3^ | 3.83x10^3^ | 3.84x10^3^ | 3.94x10^3^ |
|  |  | Maximum | 5.35x10^4^ | 5.33x10^4^ | 5.35x10^4^ | 5.25x10^4^ | 5.23x10^4^ | 5.35x10^4^ |
|  |  | Mean | 3.18x10^4^ | 3.18x10^4^ | 3.17x10^4^ | 3.12x10^4^ | 3.13x10^4^ | 3.17x10^4^ |
|  |  | Median | 3.24x10^4^ | 3.23x10^4^ | 3.23x10^4^ | 3.18x10^4^ | 3.19x10^4^ | 3.24x10^4^ |
|  | 4 | Minimum | 3.29x10^3^ | 3.28x10^3^ | 3.29x10^3^ | 3.19x10^3^ | 3.20x10^3^ | 3.29x10^3^ |
|  |  | Maximum | 5.67x10^4^ | 4.02x10^4^ | 5.67x10^4^ | 5.55x10^4^ | 5.50x10^4^ | 5.67x10^4^ |
|  |  | Mean | 3.14x10^4^ | 3.10x10^4^ | 3.14x10^4^ | 3.08x10^4^ | 3.04x10^4^ | 3.14x10^4^ |
|  |  | Median | 3.29x10^4^ | 3.12x10^4^ | 3.29x10^4^ | 3.23x10^4^ | 3.07x10^4^ | 3.29x10^4^ |

Supplementary table S4. The distribution of median micro-averaged MuMCC scores for each DNA and RNA population type from each aligner tested.

|  |  |  |  |  |  |  |  |  |
| --- | --- | --- | --- | --- | --- | --- | --- | --- |
|  | | | BWA-MEM | GEM3 | Bowtie2-Local | Bowtie2-Global | HiSAT2 | SMALT |
| RNA | High | Minimum | 0.009 | 0.010 | 0.008 | 0.010 | 0.015 | 0.008 |
|  |  | 1^st^ Quartile | 0.115 | 0.151 | 0.102 | 0.099 | 0.139 | 0.102 |
|  |  | Median | 0.347 | 0.382 | 0.321 | 0.254 | 0.324 | 0.321 |
|  |  | Mean | 0.362 | 0.389 | 0.346 | 0.213 | 0.362 | 0.347 |
|  |  | 3^rd^ Quartile | 0.577 | 0.577 | 0.577 | 0.288 | 0.577 | 0.577 |
|  |  | Maximum | 0.816 | 0.855 | 0.816 | 0.500 | 0.855 | 0.816 |
|  | Medium | Minimum | 0.009 | 0.007 | 0.007 | 0.008 | 0.007 | 0.006 |
|  |  | 1^st^ Quartile | 0.053 | 0.071 | 0.051 | 0.050 | 0.069 | 0.051 |
|  |  | Median | 0.103 | 0.103 | 0.103 | 0.097 | 0.103 | 0.103 |
|  |  | Mean | 0.254 | 0.262 | 0.248 | 0.146 | 0.259 | 0.249 |
|  |  | 3^rd^ Quartile | 0.539 | 0.539 | 0.512 | 0.272 | 0.539 | 0.539 |
|  |  | Maximum | 0.707 | 0.707 | 0.707 | 0.367 | 0.707 | 0.707 |
|  | Low | Minimum | -0.004 | -0.005 | -0.003 | -0.004 | -0.002 | -0.003 |
|  |  | 1^st^ Quartile | 0.020 | 0.027 | 0.018 | 0.017 | 0.027 | 0.018 |
|  |  | Median | 0.101 | 0.096 | 0.095 | 0.061 | 0.101 | 0.090 |
|  |  | Mean | 0.126 | 0.131 | 0.122 | 0.072 | 0.131 | 0.122 |
|  |  | 3^rd^ Quartile | 0.250 | 0.250 | 0.250 | 0.133 | 0.250 | 0.250 |
|  |  | Maximum | 0.250 | 0.250 | 0.250 | 0.138 | 0.250 | 0.250 |
| DNA | High | Minimum | 0.008 | 0.008 | 0.008 | 0.008 | 0.013 | 0.008 |
|  |  | 1^st^ Quartile | 0.148 | 0.202 | 0.132 | 0.127 | 0.193 | 0.133 |
|  |  | Median | 0.568 | 0.581 | 0.504 | 0.288 | 0.547 | 0.511 |
|  |  | Mean | 0.453 | 0.490 | 0.404 | 0.233 | 0.463 | 0.433 |
|  |  | 3^rd^ Quartile | 0.707 | 0.707 | 0.707 | 0.299 | 0.707 | 0.707 |
|  |  | Maximum | 1.000 | 1.000 | 1.000 | 0.554 | 1.000 | 1.000 |
|  | Low | Minimum | 0.003 | 0.002 | 0.004 | 0.006 | 0.002 | 0.005 |
|  |  | 1^st^ Quartile | 0.058 | 0.093 | 0.053 | 0.052 | 0.087 | 0.053 |
|  |  | Median | 0.376 | 0.395 | 0.327 | 0.202 | 0.402 | 0.327 |
|  |  | Mean | 0.368 | 0.394 | 0.351 | 0.172 | 0.391 | 0.352 |
|  |  | 3^rd^ Quartile | 0.707 | 0.707 | 0.707 | 0.288 | 0.707 | 0.707 |
|  |  | Maximum | 0.707 | 0.729 | 0.707 | 0.301 | 0.707 | 0.707 |

Supplementary table S5. The distribution of the median micro-averaged MuMCC scores generated from testing different qScore parameters for each DNA and RNA population replicate combination following alignment of reads using GEM3.

| **Singlets** | | | | | | | | | |
| --- | --- | --- | --- | --- | --- | --- | --- | --- | --- |
|  | | | 0 | 10 | 20 | 25 | 30 | 35 | 38 |
| RNA | High | Minimum | 0.010 | 0.010 | 0.022 | 0.025 | 0.041 | 0.048 | 0.053 |
|  |  | 1^st^ Quartile | 0.054 | 0.054 | 0.084 | 0.151 | 0.229 | 0.252 | 0.201 |
|  |  | Median | 0.096 | 0.096 | 0.115 | 0.205 | 0.360 | 0.434 | 0.375 |
|  |  | Mean | 0.236 | 0.236 | 0.286 | 0.352 | 0.415 | 0.435 | 0.423 |
|  |  | 3^rd^ Quartile | 0.439 | 0.439 | 0.538 | 0.577 | 0.577 | 0.577 | 0.642 |
|  |  | Maximum | 0.642 | 0.642 | 0.707 | 0.707 | 0.756 | 0.756 | 0.855 |
|  | Medium | Minimum | 0.007 | 0.007 | 0.012 | 0.015 | 0.027 | 0.021 | 0.029 |
|  |  | 1^st^ Quartile | 0.021 | 0.021 | 0.022 | 0.047 | 0.083 | 0.098 | 0.093 |
|  |  | Median | 0.034 | 0.034 | 0.051 | 0.084 | 0.101 | 0.103 | 0.102 |
|  |  | Mean | 0.215 | 0.215 | 0.234 | 0.258 | 0.271 | 0.279 | 0.264 |
|  |  | 3^rd^ Quartile | 0.408 | 0.408 | 0.512 | 0.538 | 0.538 | 0.538 | 0.512 |
|  |  | Maximum | 0.707 | 0.707 | 0.707 | 0.707 | 0.707 | 0.707 | 0.707 |
|  | Low | Minimum | -0.005 | -0.005 | -0.004 | 0.003 | 0.008 | 0.011 | 0.004 |
|  |  | 1^st^ Quartile | 0.003 | 0.003 | 0.004 | 0.015 | 0.037 | 0.069 | 0.047 |
|  |  | Median | 0.014 | 0.014 | 0.016 | 0.036 | 0.082 | 0.108 | 0.108 |
|  |  | Mean | 0.092 | 0.092 | 0.102 | 0.119 | 0.137 | 0.148 | 0.139 |
|  |  | 3^rd^ Quartile | 0.209 | 0.209 | 0.250 | 0.250 | 0.250 | 0.250 | 0.250 |
|  |  | Maximum | 0.250 | 0.250 | 0.250 | 0.250 | 0.250 | 0.250 | 0.250 |
| DNA | High | Minimum | 0.008 | 0.008 | 0.019 | 0.033 | 0.056 | 0.062 | 0.074 |
|  |  | 1^st^ Quartile | 0.053 | 0.053 | 0.073 | 0.143 | 0.356 | 0.547 | 0.322 |
|  |  | Median | 0.071 | 0.071 | 0.099 | 0.185 | 0.435 | 0.707 | 0.642 |
|  |  | Mean | 0.258 | 0.258 | 0.311 | 0.393 | 0.510 | 0.600 | 0.533 |
|  |  | 3^rd^ Quartile | 0.500 | 0.500 | 0.707 | 0.707 | 0.707 | 0.707 | 0.707 |
|  |  | Maximum | 0.447 | 0.447 | 0.634 | 0.564 | 0.351 | 0.160 | 0.385 |
|  | Low | Minimum | 0.002 | 0.002 | 0.006 | 0.015 | 0.025 | 0.032 | 0.021 |
|  |  | 1^st^ Quartile | 0.012 | 0.012 | 0.017 | 0.052 | 0.125 | 0.275 | 0.174 |
|  |  | Median | 0.018 | 0.018 | 0.042 | 0.114 | 0.326 | 0.455 | 0.417 |
|  |  | Mean | 0.209 | 0.209 | 0.271 | 0.339 | 0.415 | 0.471 | 0.438 |
|  |  | 3^rd^ Quartile | 0.427 | 0.427 | 0.707 | 0.707 | 0.707 | 0.707 | 0.707 |
|  |  | Maximum | 0.707 | 0.707 | 0.707 | 0.707 | 0.707 | 0.707 | 0.707 |

| **Duplicates** | | | | | | | | | | |
| --- | --- | --- | --- | --- | --- | --- | --- | --- | --- | --- |
|  | | | 0 | 10 | 20 | 25 | 30 | 35 | 38 |  |
| RNA | High | Minimum | 0.013 | 0.013 | 0.028 | 0.035 | 0.056 | 0.068 | 0.078 |  |
|  |  | 1^st^ Quartile | 0.065 | 0.065 | 0.092 | 0.173 | 0.296 | 0.340 | 0.369 |  |
|  |  | Median | 0.109 | 0.109 | 0.124 | 0.232 | 0.377 | 0.475 | 0.543 |  |
|  |  | Mean | 0.265 | 0.265 | 0.303 | 0.371 | 0.437 | 0.467 | 0.504 |  |
|  |  | 3^rd^ Quartile | 0.500 | 0.500 | 0.577 | 0.590 | 0.642 | 0.642 | 0.707 |  |
|  |  | Maximum | 0.707 | 0.707 | 0.707 | 0.707 | 0.786 | 0.816 | 0.816 |  |
|  | Medium | Minimum | 0.009 | 0.009 | 0.008 | 0.018 | 0.036 | 0.025 | 0.032 |  |
|  |  | 1^st^ Quartile | 0.017 | 0.017 | 0.022 | 0.046 | 0.097 | 0.162 | 0.169 |  |
|  |  | Median | 0.024 | 0.024 | 0.040 | 0.091 | 0.173 | 0.197 | 0.192 |  |
|  |  | Mean | 0.254 | 0.254 | 0.286 | 0.322 | 0.357 | 0.378 | 0.380 |  |
|  |  | 3^rd^ Quartile | 0.555 | 0.555 | 0.707 | 0.707 | 0.707 | 0.707 | 0.707 |  |
|  |  | Maximum | 0.707 | 0.707 | 0.707 | 0.707 | 0.707 | 0.707 | 0.707 |  |
|  | Low | Minimum | -0.013 | -0.013 | -0.011 | -0.010 | -0.008 | -0.002 | -0.010 |  |
|  |  | 1^st^ Quartile | -0.008 | -0.008 | -0.008 | -0.005 | -0.002 | -0.002 | -0.003 |  |
|  |  | Median | -0.001 | -0.001 | -0.006 | -0.003 | -0.001 | 0.002 | -0.002 |  |
|  |  | Mean | -0.001 | -0.001 | -0.003 | -0.002 | 0.000 | 0.005 | -0.002 |  |
|  |  | 3^rd^ Quartile | 0.006 | 0.006 | 0.000 | -0.001 | -0.001 | 0.010 | -0.001 |  |
|  |  | Maximum | 0.018 | 0.018 | 0.019 | 0.022 | 0.027 | 0.023 | 0.001 |  |
| DNA | High | Minimum | 0.011 | 0.011 | 0.023 | 0.042 | 0.077 | 0.094 | 0.137 |  |
|  |  | 1^st^ Quartile | 0.057 | 0.057 | 0.083 | 0.153 | 0.398 | 0.577 | 0.577 |  |
|  |  | Median | 0.076 | 0.076 | 0.101 | 0.195 | 0.494 | 0.707 | 0.707 |  |
|  |  | Mean | 0.272 | 0.272 | 0.325 | 0.407 | 0.535 | 0.645 | 0.608 |  |
|  |  | 3^rd^ Quartile | 0.538 | 0.538 | 0.707 | 0.707 | 0.707 | 0.707 | 0.707 |  |
|  |  | Maximum | 0.707 | 0.707 | 0.707 | 0.711 | 1.000 | 0.933 | 0.854 |  |
|  | Low | Minimum | 0.001 | 0.001 | 0.004 | 0.012 | 0.030 | 0.036 | 0.038 |  |
|  |  | 1^st^ Quartile | 0.011 | 0.011 | 0.015 | 0.036 | 0.121 | 0.408 | 0.370 |  |
|  |  | Median | 0.019 | 0.019 | 0.031 | 0.088 | 0.288 | 0.408 | 0.408 |  |
|  |  | Mean | 0.227 | 0.227 | 0.280 | 0.340 | 0.409 | 0.495 | 0.490 |  |
|  |  | 3^rd^ Quartile | 0.500 | 0.500 | 0.707 | 0.707 | 0.707 | 0.707 | 0.707 |  |
|  |  | Maximum | 0.707 | 0.707 | 0.707 | 0.707 | 0.707 | 0.707 | 0.707 |  |

| **Triplicates** | | | | | | | | | | |
| --- | --- | --- | --- | --- | --- | --- | --- | --- | --- | --- |
|  | | | 0 | 10 | 20 | 25 | 30 | 35 | 38 |  |
| RNA | High | Minimum | 0.015 | 0.015 | 0.030 | 0.039 | 0.063 | 0.078 | 0.085 |  |
|  |  | 1^st^ Quartile | 0.070 | 0.070 | 0.090 | 0.169 | 0.299 | 0.347 | 0.468 |  |
|  |  | Median | 0.114 | 0.114 | 0.133 | 0.252 | 0.412 | 0.500 | 0.577 |  |
|  |  | Mean | 0.276 | 0.276 | 0.314 | 0.383 | 0.455 | 0.486 | 0.540 |  |
|  |  | 3^rd^ Quartile | 0.500 | 0.500 | 0.577 | 0.618 | 0.707 | 0.707 | 0.707 |  |
|  |  | Maximum | 0.707 | 0.707 | 0.707 | 0.707 | 0.816 | 0.816 | 0.894 |  |
|  | Medium | Minimum | 0.003 | 0.003 | 0.008 | 0.010 | 0.033 | 0.027 | 0.049 |  |
|  |  | 1^st^ Quartile | 0.018 | 0.018 | 0.021 | 0.046 | 0.136 | 0.273 | 0.288 |  |
|  |  | Median | 0.027 | 0.027 | 0.046 | 0.109 | 0.246 | 0.333 | 0.333 |  |
|  |  | Mean | 0.265 | 0.265 | 0.297 | 0.340 | 0.394 | 0.442 | 0.455 |  |
|  |  | 3^rd^ Quartile | 0.610 | 0.610 | 0.707 | 0.707 | 0.707 | 0.707 | 0.707 |  |
|  |  | Maximum | 0.707 | 0.707 | 0.707 | 0.707 | 0.707 | 0.707 | 0.707 |  |
|  | Low | Minimum | -0.016 | -0.016 | -0.012 | -0.010 | -0.006 | -0.004 | -0.007 |  |
|  |  | 1^st^ Quartile | -0.009 | -0.009 | -0.008 | -0.005 | -0.002 | -0.003 | -0.002 |  |
|  |  | Median | -0.001 | -0.001 | -0.005 | -0.003 | -0.001 | -0.001 | -0.001 |  |
|  |  | Mean | -0.002 | -0.002 | -0.004 | -0.003 | 0.000 | 0.000 | -0.002 |  |
|  |  | 3^rd^ Quartile | 0.005 | 0.005 | -0.001 | -0.002 | -0.001 | 0.000 | 0.000 |  |
|  |  | Maximum | 0.020 | 0.020 | 0.020 | 0.017 | 0.021 | 0.012 | 0.000 |  |
| DNA | High | Minimum | 0.011 | 0.011 | 0.025 | 0.047 | 0.091 | 0.119 | 0.211 |  |
|  |  | 1^st^ Quartile | 0.060 | 0.060 | 0.086 | 0.155 | 0.408 | 0.577 | 0.577 |  |
|  |  | Median | 0.079 | 0.079 | 0.103 | 0.205 | 0.530 | 0.707 | 0.707 |  |
|  |  | Mean | 0.279 | 0.279 | 0.332 | 0.415 | 0.547 | 0.657 | 0.634 |  |
|  |  | 3^rd^ Quartile | 0.577 | 0.577 | 0.707 | 0.707 | 0.707 | 0.707 | 0.707 |  |
|  |  | Maximum | 0.707 | 0.707 | 0.707 | 0.756 | 1.000 | 0.913 | 0.897 |  |
|  | Low | Minimum | -0.002 | -0.002 | -0.001 | 0.008 | 0.027 | 0.050 | 0.042 |  |
|  |  | 1^st^ Quartile | 0.011 | 0.011 | 0.016 | 0.040 | 0.139 | 0.408 | 0.408 |  |
|  |  | Median | 0.021 | 0.021 | 0.033 | 0.095 | 0.333 | 0.408 | 0.408 |  |
|  |  | Mean | 0.235 | 0.235 | 0.286 | 0.347 | 0.418 | 0.503 | 0.510 |  |
|  |  | 3^rd^ Quartile | 0.538 | 0.538 | 0.707 | 0.707 | 0.707 | 0.707 | 0.707 |  |
|  |  | Maximum | 0.707 | 0.707 | 0.707 | 0.707 | 0.707 | 0.707 | 0.707 |  |

| **Quadruplicates** | | | | | | | | | |
| --- | --- | --- | --- | --- | --- | --- | --- | --- | --- |
|  | | | 0 | 10 | 20 | 25 | 30 | 35 | 38 |
| RNA | High | Minimum | 0.017 | 0.017 | 0.031 | 0.044 | 0.071 | 0.089 | 0.105 |
|  |  | 1^st^ Quartile | 0.072 | 0.072 | 0.093 | 0.174 | 0.308 | 0.347 | 0.480 |
|  |  | Median | 0.119 | 0.119 | 0.141 | 0.262 | 0.454 | 0.529 | 0.577 |
|  |  | Mean | 0.279 | 0.279 | 0.316 | 0.387 | 0.462 | 0.495 | 0.564 |
|  |  | 3^rd^ Quartile | 0.500 | 0.500 | 0.577 | 0.632 | 0.707 | 0.707 | 0.707 |
|  |  | Maximum | 0.707 | 0.707 | 0.707 | 0.707 | 0.816 | 0.816 | 0.894 |
|  | Medium | Minimum | 0.005 | 0.005 | 0.009 | 0.012 | 0.041 | 0.023 | 0.067 |
|  |  | 1^st^ Quartile | 0.019 | 0.019 | 0.023 | 0.051 | 0.165 | 0.408 | 0.408 |
|  |  | Median | 0.029 | 0.029 | 0.051 | 0.121 | 0.288 | 0.408 | 0.408 |
|  |  | Mean | 0.269 | 0.269 | 0.303 | 0.349 | 0.418 | 0.493 | 0.513 |
|  |  | 3^rd^ Quartile | 0.610 | 0.610 | 0.707 | 0.707 | 0.707 | 0.707 | 0.707 |
|  |  | Maximum | 0.707 | 0.707 | 0.707 | 0.707 | 0.707 | 0.707 | 0.707 |
|  | Low | Minimum | -0.015 | -0.015 | -0.012 | -0.009 | -0.006 | -0.011 | -0.005 |
|  |  | 1^st^ Quartile | -0.009 | -0.009 | -0.008 | -0.005 | -0.002 | -0.002 | -0.001 |
|  |  | Median | -0.001 | -0.001 | -0.005 | -0.003 | -0.001 | -0.001 | 0.000 |
|  |  | Mean | -0.001 | -0.001 | -0.004 | -0.002 | 0.000 | -0.001 | -0.001 |
|  |  | 3^rd^ Quartile | 0.005 | 0.005 | -0.001 | -0.002 | -0.001 | 0.000 | 0.000 |
|  |  | Maximum | 0.022 | 0.022 | 0.020 | 0.016 | 0.019 | 0.002 | 0.000 |
| DNA | High | Minimum | 0.013 | 0.013 | 0.027 | 0.051 | 0.100 | 0.151 | 0.311 |
|  |  | 1^st^ Quartile | 0.061 | 0.061 | 0.088 | 0.164 | 0.408 | 0.577 | 0.577 |
|  |  | Median | 0.081 | 0.081 | 0.109 | 0.214 | 0.539 | 0.707 | 0.707 |
|  |  | Mean | 0.282 | 0.282 | 0.337 | 0.418 | 0.555 | 0.667 | 0.649 |
|  |  | 3^rd^ Quartile | 0.577 | 0.577 | 0.707 | 0.707 | 0.707 | 0.707 | 0.707 |
|  |  | Maximum | 0.707 | 0.707 | 0.707 | 0.756 | 1.000 | 0.913 | 0.913 |
|  | Low | Minimum | -0.006 | -0.006 | -0.006 | 0.008 | 0.027 | 0.058 | 0.056 |
|  |  | 1^st^ Quartile | 0.011 | 0.011 | 0.016 | 0.044 | 0.153 | 0.408 | 0.408 |
|  |  | Median | 0.022 | 0.022 | 0.036 | 0.103 | 0.333 | 0.408 | 0.408 |
|  |  | Mean | 0.236 | 0.236 | 0.288 | 0.348 | 0.424 | 0.507 | 0.522 |
|  |  | 3^rd^ Quartile | 0.577 | 0.577 | 0.707 | 0.707 | 0.707 | 0.707 | 0.707 |
|  |  | Maximum | 0.707 | 0.707 | 0.707 | 0.707 | 0.707 | 0.707 | 0.707 |

Supplementary table S6. The distribution of the median micro-averaged MuMCC scores generated from testing different read length parameters for each DNA and RNA population replicate combination following alignment of reads using GEM3 and chosen qScore parameter.

| **Singlets** | | | | | | | | | |
| --- | --- | --- | --- | --- | --- | --- | --- | --- | --- |
|  | | | 70 | 80 | 90 | 100 | 110 | 120 | 130 |
| RNA | High | Minimum | 0.053 | 0.048 | 0.048 | 0.050 | 0.050 | 0.054 | 0.055 |
|  |  | 1^st^ Quartile | 0.265 | 0.253 | 0.248 | 0.242 | 0.232 | 0.234 | 0.210 |
|  |  | Median | 0.469 | 0.447 | 0.424 | 0.414 | 0.427 | 0.380 | 0.354 |
|  |  | Mean | 0.448 | 0.445 | 0.436 | 0.435 | 0.431 | 0.431 | 0.423 |
|  |  | 3^rd^ Quartile | 0.577 | 0.577 | 0.577 | 0.577 | 0.577 | 0.642 | 0.577 |
|  |  | Maximum | 0.756 | 0.756 | 0.756 | 0.756 | 0.756 | 0.756 | 0.756 |
|  | Medium | Minimum | 0.039 | 0.034 | 0.034 | 0.029 | 0.024 | 0.023 | 0.021 |
|  |  | 1^st^ Quartile | 0.099 | 0.098 | 0.098 | 0.097 | 0.097 | 0.095 | 0.094 |
|  |  | Median | 0.103 | 0.103 | 0.103 | 0.103 | 0.103 | 0.103 | 0.103 |
|  |  | Mean | 0.282 | 0.281 | 0.278 | 0.282 | 0.278 | 0.275 | 0.275 |
|  |  | 3^rd^ Quartile | 0.577 | 0.577 | 0.539 | 0.539 | 0.539 | 0.539 | 0.539 |
|  |  | Maximum | 0.707 | 0.707 | 0.707 | 0.707 | 0.707 | 0.707 | 0.707 |
|  | Low | Minimum | 0.020 | 0.012 | 0.011 | 0.017 | 0.018 | 0.015 | 0.022 |
|  |  | 1^st^ Quartile | 0.067 | 0.069 | 0.069 | 0.068 | 0.061 | 0.062 | 0.057 |
|  |  | Median | 0.101 | 0.101 | 0.117 | 0.109 | 0.109 | 0.109 | 0.109 |
|  |  | Mean | 0.148 | 0.147 | 0.148 | 0.149 | 0.149 | 0.146 | 0.146 |
|  |  | 3^rd^ Quartile | 0.250 | 0.250 | 0.250 | 0.250 | 0.250 | 0.250 | 0.250 |
|  |  | Maximum | 0.250 | 0.250 | 0.250 | 0.250 | 0.250 | 0.250 | 0.250 |
| DNA | High | Minimum | 0.069 | 0.066 | 0.065 | 0.062 | 0.067 | 0.066 | 0.070 |
|  |  | 1^st^ Quartile | 0.590 | 0.577 | 0.547 | 0.553 | 0.541 | 0.507 | 0.526 |
|  |  | Median | 0.707 | 0.707 | 0.707 | 0.707 | 0.707 | 0.699 | 0.706 |
|  |  | Mean | 0.619 | 0.604 | 0.599 | 0.601 | 0.601 | 0.588 | 0.588 |
|  |  | 3^rd^ Quartile | 0.707 | 0.707 | 0.707 | 0.707 | 0.707 | 0.707 | 0.707 |
|  |  | Maximum | 1.000 | 0.933 | 0.854 | 1.000 | 1.000 | 0.854 | 1.000 |
|  | Low | Minimum | 0.039 | 0.039 | 0.032 | 0.032 | 0.036 | 0.040 | 0.045 |
|  |  | 1^st^ Quartile | 0.296 | 0.282 | 0.281 | 0.260 | 0.245 | 0.216 | 0.208 |
|  |  | Median | 0.455 | 0.455 | 0.455 | 0.455 | 0.433 | 0.433 | 0.433 |
|  |  | Mean | 0.483 | 0.485 | 0.477 | 0.469 | 0.467 | 0.459 | 0.459 |
|  |  | 3^rd^ Quartile | 0.707 | 0.707 | 0.707 | 0.707 | 0.707 | 0.707 | 0.707 |
|  |  | Maximum | 0.707 | 0.707 | 0.707 | 0.707 | 0.707 | 0.707 | 0.707 |

| **Duplicates** | | | | | | | | | | |
| --- | --- | --- | --- | --- | --- | --- | --- | --- | --- | --- |
|  | | | 70 | 80 | 90 | 100 | 110 | 120 | 130 |  |
| RNA | High | Minimum | 0.084 | 0.084 | 0.078 | 0.081 | 0.088 | 0.086 | 0.101 |  |
|  |  | 1^st^ Quartile | 0.480 | 0.466 | 0.436 | 0.400 | 0.344 | 0.305 | 0.256 |  |
|  |  | Median | 0.577 | 0.541 | 0.543 | 0.547 | 0.513 | 0.456 | 0.417 |  |
|  |  | Mean | 0.524 | 0.525 | 0.515 | 0.515 | 0.503 | 0.482 | 0.466 |  |
|  |  | 3^rd^ Quartile | 0.707 | 0.707 | 0.707 | 0.707 | 0.707 | 0.707 | 0.707 |  |
|  |  | Maximum | 0.816 | 0.816 | 0.816 | 0.816 | 0.816 | 0.816 | 0.816 |  |
|  | Medium | Minimum | 0.040 | 0.036 | 0.040 | 0.033 | 0.034 | 0.028 | 0.025 |  |
|  |  | 1^st^ Quartile | 0.165 | 0.158 | 0.165 | 0.161 | 0.159 | 0.160 | 0.153 |  |
|  |  | Median | 0.203 | 0.197 | 0.197 | 0.197 | 0.197 | 0.197 | 0.187 |  |
|  |  | Mean | 0.381 | 0.378 | 0.379 | 0.378 | 0.378 | 0.376 | 0.374 |  |
|  |  | 3^rd^ Quartile | 0.707 | 0.707 | 0.707 | 0.707 | 0.707 | 0.707 | 0.707 |  |
|  |  | Maximum | 0.707 | 0.707 | 0.707 | 0.707 | 0.707 | 0.707 | 0.707 |  |
|  | Low | Minimum | -0.002 | -0.002 | -0.002 | -0.002 | -0.002 | -0.002 | -0.002 |  |
|  |  | 1^st^ Quartile | -0.001 | -0.001 | -0.001 | -0.001 | -0.002 | -0.001 | -0.002 |  |
|  |  | Median | 0.003 | 0.003 | 0.003 | 0.003 | 0.010 | 0.001 | 0.016 |  |
|  |  | Mean | 0.006 | 0.005 | 0.004 | 0.004 | 0.009 | 0.001 | 0.008 |  |
|  |  | 3^rd^ Quartile | 0.010 | 0.009 | 0.008 | 0.008 | 0.017 | 0.006 | 0.016 |  |
|  |  | Maximum | 0.023 | 0.015 | 0.015 | 0.014 | 0.018 | 0.006 | 0.020 |  |
| DNA | High | Minimum | 0.096 | 0.094 | 0.098 | 0.096 | 0.101 | 0.098 | 0.103 |  |
|  |  | 1^st^ Quartile | 0.577 | 0.577 | 0.577 | 0.577 | 0.577 | 0.577 | 0.577 |  |
|  |  | Median | 0.707 | 0.707 | 0.707 | 0.707 | 0.707 | 0.707 | 0.707 |  |
|  |  | Mean | 0.654 | 0.640 | 0.640 | 0.659 | 0.648 | 0.633 | 0.644 |  |
|  |  | 3^rd^ Quartile | 0.707 | 0.707 | 0.707 | 0.707 | 0.707 | 0.707 | 0.707 |  |
|  |  | Maximum | 0.933 | 0.857 | 0.892 | 0.933 | 0.933 | 0.807 | 0.933 |  |
|  | Low | Minimum | 0.050 | 0.045 | 0.036 | 0.036 | 0.043 | 0.041 | 0.055 |  |
|  |  | 1^st^ Quartile | 0.408 | 0.408 | 0.408 | 0.408 | 0.399 | 0.371 | 0.371 |  |
|  |  | Median | 0.408 | 0.408 | 0.408 | 0.408 | 0.408 | 0.408 | 0.408 |  |
|  |  | Mean | 0.496 | 0.501 | 0.499 | 0.495 | 0.496 | 0.493 | 0.488 |  |
|  |  | 3^rd^ Quartile | 0.707 | 0.707 | 0.707 | 0.707 | 0.707 | 0.707 | 0.707 |  |
|  |  | Maximum | 0.707 | 0.707 | 0.707 | 0.707 | 0.707 | 0.707 | 0.707 |  |

| **Triplicates** | | | | | | | | | |
| --- | --- | --- | --- | --- | --- | --- | --- | --- | --- |
|  | | | 70 | 80 | 90 | 100 | 110 | 120 | 130 |
| RNA | High | Minimum | 0.112 | 0.114 | 0.098 | 0.105 | 0.115 | 0.085 | 0.105 |
|  |  | 1^st^ Quartile | 0.481 | 0.500 | 0.468 | 0.456 | 0.467 | 0.409 | 0.373 |
|  |  | Median | 0.577 | 0.577 | 0.577 | 0.577 | 0.541 | 0.540 | 0.481 |
|  |  | Mean | 0.553 | 0.556 | 0.544 | 0.549 | 0.541 | 0.524 | 0.515 |
|  |  | 3^rd^ Quartile | 0.707 | 0.707 | 0.707 | 0.707 | 0.707 | 0.707 | 0.707 |
|  |  | Maximum | 0.816 | 0.816 | 0.816 | 0.816 | 0.816 | 0.816 | 0.894 |
|  | Medium | Minimum | 0.035 | 0.035 | 0.036 | 0.038 | 0.038 | 0.032 | 0.027 |
|  |  | 1^st^ Quartile | 0.284 | 0.284 | 0.273 | 0.284 | 0.273 | 0.273 | 0.288 |
|  |  | Median | 0.333 | 0.333 | 0.333 | 0.333 | 0.333 | 0.311 | 0.311 |
|  |  | Mean | 0.442 | 0.441 | 0.442 | 0.441 | 0.441 | 0.439 | 0.446 |
|  |  | 3^rd^ Quartile | 0.707 | 0.707 | 0.707 | 0.707 | 0.707 | 0.707 | 0.707 |
|  |  | Maximum | 0.707 | 0.707 | 0.707 | 0.707 | 0.707 | 0.707 | 0.707 |
|  | Low | Minimum | -0.003 | -0.003 | -0.003 | -0.003 | -0.003 | -0.004 | -0.003 |
|  |  | 1^st^ Quartile | -0.003 | -0.002 | -0.002 | -0.002 | -0.003 | -0.004 | -0.003 |
|  |  | Median | -0.001 | -0.001 | -0.001 | -0.001 | -0.001 | -0.002 | -0.001 |
|  |  | Mean | 0.002 | 0.000 | 0.000 | 0.000 | 0.000 | -0.002 | 0.000 |
|  |  | 3^rd^ Quartile | 0.012 | 0.001 | 0.001 | 0.001 | 0.006 | -0.001 | 0.002 |
|  |  | Maximum | 0.012 | 0.007 | 0.007 | 0.006 | 0.006 | -0.001 | 0.006 |
| DNA | High | Minimum | 0.124 | 0.119 | 0.125 | 0.127 | 0.134 | 0.134 | 0.136 |
|  |  | 1^st^ Quartile | 0.577 | 0.577 | 0.577 | 0.577 | 0.577 | 0.577 | 0.577 |
|  |  | Median | 0.707 | 0.707 | 0.707 | 0.707 | 0.707 | 0.707 | 0.707 |
|  |  | Mean | 0.663 | 0.651 | 0.655 | 0.673 | 0.655 | 0.645 | 0.661 |
|  |  | 3^rd^ Quartile | 0.707 | 0.707 | 0.707 | 0.735 | 0.707 | 0.707 | 0.707 |
|  |  | Maximum | 0.892 | 0.892 | 0.892 | 0.913 | 0.866 | 0.852 | 0.866 |
|  | Low | Minimum | 0.056 | 0.057 | 0.050 | 0.050 | 0.050 | 0.052 | 0.062 |
|  |  | 1^st^ Quartile | 0.408 | 0.408 | 0.408 | 0.408 | 0.408 | 0.408 | 0.408 |
|  |  | Median | 0.408 | 0.408 | 0.408 | 0.408 | 0.408 | 0.408 | 0.408 |
|  |  | Mean | 0.503 | 0.502 | 0.502 | 0.505 | 0.503 | 0.505 | 0.499 |
|  |  | 3^rd^ Quartile | 0.707 | 0.707 | 0.707 | 0.707 | 0.707 | 0.707 | 0.707 |
|  |  | Maximum | 0.707 | 0.707 | 0.707 | 0.707 | 0.707 | 0.707 | 0.707 |

| **Quadruplicates** | | | | | | | | | | |
| --- | --- | --- | --- | --- | --- | --- | --- | --- | --- | --- |
|  | | | 70 | 80 | 90 | 100 | 110 | 120 | 130 |  |
| RNA | High | Minimum | 0.132 | 0.135 | 0.115 | 0.128 | 0.142 | 0.105 | 0.133 |  |
|  |  | 1^st^ Quartile | 0.481 | 0.500 | 0.481 | 0.481 | 0.522 | 0.489 | 0.430 |  |
|  |  | Median | 0.577 | 0.596 | 0.577 | 0.583 | 0.577 | 0.577 | 0.521 |  |
|  |  | Mean | 0.571 | 0.579 | 0.569 | 0.569 | 0.564 | 0.552 | 0.543 |  |
|  |  | 3^rd^ Quartile | 0.707 | 0.707 | 0.707 | 0.707 | 0.707 | 0.707 | 0.707 |  |
|  |  | Maximum | 0.816 | 0.816 | 0.816 | 0.816 | 0.816 | 0.816 | 0.894 |  |
|  | Medium | Minimum | 0.033 | 0.034 | 0.034 | 0.035 | 0.036 | 0.036 | 0.023 |  |
|  |  | 1^st^ Quartile | 0.408 | 0.408 | 0.408 | 0.408 | 0.408 | 0.408 | 0.408 |  |
|  |  | Median | 0.408 | 0.408 | 0.408 | 0.408 | 0.408 | 0.408 | 0.408 |  |
|  |  | Mean | 0.494 | 0.494 | 0.492 | 0.492 | 0.494 | 0.489 | 0.497 |  |
|  |  | 3^rd^ Quartile | 0.707 | 0.707 | 0.707 | 0.707 | 0.707 | 0.707 | 0.707 |  |
|  |  | Maximum | 0.707 | 0.707 | 0.707 | 0.707 | 0.707 | 0.707 | 0.707 |  |
|  | Low | Minimum | -0.003 | -0.003 | -0.003 | -0.003 | -0.002 | -0.011 | -0.002 |  |
|  |  | 1^st^ Quartile | -0.003 | -0.002 | -0.002 | -0.002 | -0.002 | -0.005 | -0.002 |  |
|  |  | Median | -0.001 | -0.001 | -0.001 | -0.001 | -0.001 | -0.002 | -0.001 |  |
|  |  | Mean | -0.001 | -0.001 | -0.001 | -0.001 | -0.001 | -0.004 | 0.000 |  |
|  |  | 3^rd^ Quartile | 0.001 | 0.000 | 0.000 | 0.000 | 0.000 | -0.001 | 0.002 |  |
|  |  | Maximum | 0.001 | 0.001 | 0.001 | 0.000 | 0.000 | -0.001 | 0.002 |  |
| DNA | High | Minimum | 0.152 | 0.151 | 0.154 | 0.155 | 0.171 | 0.175 | 0.184 |  |
|  |  | 1^st^ Quartile | 0.577 | 0.577 | 0.577 | 0.577 | 0.577 | 0.577 | 0.577 |  |
|  |  | Median | 0.707 | 0.707 | 0.707 | 0.707 | 0.707 | 0.707 | 0.707 |  |
|  |  | Mean | 0.669 | 0.661 | 0.664 | 0.686 | 0.668 | 0.652 | 0.672 |  |
|  |  | 3^rd^ Quartile | 0.707 | 0.707 | 0.707 | 0.707 | 0.707 | 0.707 | 0.707 |  |
|  |  | Maximum | 0.913 | 0.913 | 0.913 | 0.913 | 0.881 | 0.870 | 0.870 |  |
|  | Low | Minimum | 0.063 | 0.063 | 0.061 | 0.063 | 0.062 | 0.058 | 0.078 |  |
|  |  | 1^st^ Quartile | 0.408 | 0.408 | 0.408 | 0.408 | 0.408 | 0.408 | 0.408 |  |
|  |  | Median | 0.408 | 0.408 | 0.408 | 0.408 | 0.408 | 0.408 | 0.408 |  |
|  |  | Mean | 0.504 | 0.504 | 0.503 | 0.510 | 0.508 | 0.510 | 0.508 |  |
|  |  | 3^rd^ Quartile | 0.707 | 0.707 | 0.707 | 0.707 | 0.707 | 0.707 | 0.707 |  |
|  |  | Maximum | 0.707 | 0.707 | 0.707 | 0.707 | 0.707 | 0.707 | 0.707 |  |

Supplementary table S7. The distribution of the median MuMCC scores generated from testing different percentage frequency threshold cut-offs for each DNA and RNA replicate combination following alignment of reads using GEM3 and chosen qScore and read length parameters. The MuMCC scores representing the suggested percentage frequency cut-offs are highlighted in green while the MuMCC scores representing percentage frequency cut-offs which could be applied at the cost of increased error are highlighted in amber.

|  |  |  | **Percentage frequency threshold cut-offs tested** | | | | | | | | | | | | | | | | | | |
| --- | --- | --- | --- | --- | --- | --- | --- | --- | --- | --- | --- | --- | --- | --- | --- | --- | --- | --- | --- | --- | --- |
|  | | | 0.01% | 0.02% | 0.03% | 0.04% | 0.05% | 0.06% | 0.07% | 0.08% | 0.09% | 0.10% | 0.20% | 0.30% | 0.40% | 0.50% | 0.60% | 0.70% | 0.80% | 0.90% | 1.00% |
| Singlet | RNA | *High* | 0.054 | 0.094 | 0.142 | 0.203 | 0.264 | 0.341 | 0.384 | 0.469 | 0.442 | 0.397 | 0.756 | 0.658 | 0.577 | 0.577 | 0.577 | 0.577 | 0.577 | 0.707 | 0.707 |
|  |  | *Medium* | 0.039 | 0.054 | 0.074 | 0.090 | 0.099 | 0.099 | 0.101 | 0.102 | 0.103 | 0.103 | 0.163 | 0.198 | 0.320 | 0.538 | 0.577 | 0.577 | 0.707 | 0.707 | 0.707 |
|  |  | *Low* | 0.025 | 0.017 | 0.022 | 0.049 | 0.066 | 0.079 | 0.082 | 0.101 | 0.101 | 0.108 | 0.223 | 0.223 | 0.223 | 0.250 | 0.250 | 0.250 | 0.250 | 0.250 | 0.250 |
|  | DNA | *High* | 0.069 | 0.182 | 0.360 | 0.581 | 0.764 | 0.709 | 0.593 | 0.642 | 0.611 | 0.577 | 1.000 | 0.707 | 0.707 | 0.707 | 0.707 | 0.707 | 0.707 | 0.707 | 0.707 |
|  |  | *Low* | 0.039 | 0.069 | 0.123 | 0.237 | 0.282 | 0.375 | 0.387 | 0.433 | 0.455 | 0.455 | 0.707 | 0.707 | 0.707 | 0.707 | 0.707 | 0.707 | 0.707 | 0.707 | 0.707 |
| Duplicate | RNA | *High* | 0.084 | 0.174 | 0.258 | 0.369 | 0.466 | 0.535 | 0.541 | 0.496 | 0.480 | 0.480 | 0.816 | 0.658 | 0.577 | 0.577 | 0.577 | 0.707 | 0.707 | 0.707 | 0.707 |
|  |  | *Medium* | 0.040 | 0.094 | 0.094 | 0.129 | 0.162 | 0.186 | 0.192 | 0.192 | 0.197 | 0.203 | 0.412 | 0.473 | 0.642 | 0.707 | 0.707 | 0.707 | 0.707 | 0.707 | 0.707 |
|  |  | *Low* | 0.010 | 0.007 | -0.002 | -0.001 | 0.000 | 0.000 | 0.000 | 0.000 | 0.000 | 0.000 | 0.000 | 0.000 | 0.000 | 0.000 | 0.000 | 0.000 | 0.000 | 0.000 | 0.000 |
|  | DNA | *High* | 0.096 | 0.344 | 0.678 | 0.840 | 0.849 | 0.707 | 0.577 | 0.577 | 0.577 | 0.577 | 0.933 | 0.707 | 0.707 | 0.707 | 0.707 | 0.707 | 0.707 | 0.707 | 0.707 |
|  |  | *Low* | 0.045 | 0.090 | 0.207 | 0.360 | 0.408 | 0.408 | 0.408 | 0.408 | 0.408 | 0.408 | 0.707 | 0.707 | 0.707 | 0.707 | 0.707 | 0.707 | 0.707 | 0.707 | 0.707 |
| Triplicate | RNA | *High* | 0.114 | 0.237 | 0.381 | 0.510 | 0.594 | 0.632 | 0.559 | 0.534 | 0.480 | 0.510 | 0.816 | 0.500 | 0.577 | 0.577 | 0.707 | 0.707 | 0.707 | 0.707 | 0.707 |
|  |  | *Medium* | 0.040 | 0.067 | 0.133 | 0.210 | 0.258 | 0.288 | 0.311 | 0.311 | 0.333 | 0.577 | 0.577 | 0.707 | 0.707 | 0.707 | 0.707 | 0.707 | 0.707 | 0.707 | 0.707 |
|  |  | *Low* | 0.006 | -0.003 | -0.001 | 0.000 | 0.000 | 0.000 | 0.000 | 0.000 | 0.000 | 0.000 | 0.000 | 0.000 | 0.000 | 0.000 | 0.000 | 0.000 | 0.000 | 0.000 | 0.000 |
|  | DNA | *High* | 0.124 | 0.502 | 0.761 | 0.891 | 0.844 | 0.642 | 0.577 | 0.577 | 0.577 | 0.577 | 0.866 | 0.707 | 0.707 | 0.707 | 0.707 | 0.707 | 0.707 | 0.707 | 0.707 |
|  |  | *Low* | 0.051 | 0.120 | 0.246 | 0.370 | 0.408 | 0.408 | 0.408 | 0.408 | 0.408 | 0.408 | 0.707 | 0.707 | 0.707 | 0.707 | 0.707 | 0.707 | 0.707 | 0.707 | 0.707 |
| Quadruplicate | RNA | *High* | 0.135 | 0.293 | 0.470 | 0.602 | 0.676 | 0.680 | 0.596 | 0.547 | 0.480 | 0.510 | 0.816 | 0.500 | 0.577 | 0.577 | 0.707 | 0.707 | 0.707 | 0.707 | 0.707 |
|  |  | *Medium* | 0.024 | 0.107 | 0.165 | 0.333 | 0.408 | 0.408 | 0.408 | 0.408 | 0.408 | 0.408 | 0.707 | 0.707 | 0.707 | 0.707 | 0.707 | 0.707 | 0.707 | 0.707 | 0.707 |
|  |  | *Low* | 0.000 | -0.003 | -0.001 | 0.000 | 0.000 | 0.000 | 0.000 | 0.000 | 0.000 | 0.000 | 0.000 | 0.000 | 0.000 | 0.000 | 0.000 | 0.000 | 0.000 | 0.000 | 0.000 |
|  | DNA | *High* | 0.155 | 0.666 | 0.881 | 0.913 | 0.866 | 0.707 | 0.577 | 0.577 | 0.577 | 0.577 | 0.866 | 0.707 | 0.707 | 0.707 | 0.707 | 0.707 | 0.707 | 0.707 | 0.707 |
|  |  | *Low* | 0.063 | 0.124 | 0.288 | 0.408 | 0.408 | 0.408 | 0.408 | 0.408 | 0.408 | 0.408 | 0.707 | 0.707 | 0.707 | 0.707 | 0.707 | 0.707 | 0.707 | 0.707 | 0.707 |
